# A systematic literature review of researchers’ and healthcare professionals’ attitudes towards the secondary use and sharing of health administrative and clinical trial data

**DOI:** 10.1186/s13643-020-01485-5

**Published:** 2020-10-12

**Authors:** Elizabeth Hutchings, Max Loomes, Phyllis Butow, Frances M. Boyle

**Affiliations:** 1 Northern Clinical School, Faculty of Medicine, University of Sydney, Sydney, Australia; 2 Department of Psychology, The University of Sydney, Sydney, NSW Australia; 3 Centre for Medical Psychology & Evidence-Based Decision-Making (CeMPED), Sydney, Australia; 4 Psycho-Oncology Co-Operative Research Group (PoCoG), The University of Sydney, Sydney, NSW Australia; 5 Patricia Ritchie Centre for Cancer Care and Research, Mater Hospital, North Sydney, Sydney, Australia

**Keywords:** Secondary data analysis, Attitudes, Clinicians, Scientists

## Abstract

**Abstract:**

A systematic literature review of researchers and healthcare professionals’ attitudes towards the secondary use and sharing of health administrative and clinical trial data was conducted using electronic data searching. Eligible articles included those reporting qualitative or quantitative original research and published in English. No restrictions were placed on publication dates, study design, or disease setting. Two authors were involved in all stages of the review process; conflicts were resolved by consensus. Data was extracted independently using a pre-piloted data extraction template. Quality and bias were assessed using the QualSyst criteria for qualitative studies. Eighteen eligible articles were identified, and articles were categorised into four key themes: barriers, facilitators, access, and ownership; 14 subthemes were identified. While respondents were generally supportive of data sharing, concerns were expressed about access to data, data storage infrastructure, and consent. Perceptions of data ownership and acknowledgement, trust, and policy frameworks influenced sharing practice, as did age, discipline, professional focus, and world region. Young researchers were less willing to share data; they were willing to share in circumstances where they were acknowledged. While there is a general consensus that increased data sharing in health is beneficial to the wider scientific community, substantial barriers remain.

**Systematic review registration:**

PROSPERO CRD42018110559

## Background

Healthcare systems generate large amounts of data; approximately 80 mB of data are generated per patient per year [1]. It is projected that this figure will continue to grow with an increasing reliance on technologies and diagnostic capabilities. Healthcare data provides an opportunity for secondary data analysis with the capacity to greatly influence medical research, service planning, and health policy.

There are many forms of data collected in the healthcare setting including administrative and clinical trial data which are the focus of this review. Administrative data collected during patients’ care in the primary, secondary, and tertiary settings can be analysed to identify systemic issues and service gaps, and used to inform improved health resourcing. Clinical trials play an essential role in furthering our understanding of disease, advancing new therapeutics, and developing improved supportive care interventions. However, clinical trials are expensive and can take several years to complete; a frequently quoted figure is that it takes 17 years for 14% of clinical research to benefit the patient [2, 3].

Those who argue for increased data sharing in healthcare suggest that it may lead to improved treatment decisions based on all available information [4, 5], improved identification of causes and clinical manifestations of disease [6], and provide increased research transparency [7]. In rare diseases, secondary data analysis may greatly accelerate the medical community’s understanding of the disease’s pathology and influence treatment.

Internationally, there are signs of movement towards greater transparency, particularly with regard to clinical research data. This change has been driven by governments [8], peak bodies [9], and clinician led initiatives [5]. One initiative led by the International Council of Medical Journal Editors (ICMJE) now requires a data sharing plan for all clinical research submitted for publication in a member scientific journal [9]. Further, international examples of data sharing can be seen in projects such as The Cancer Genome Atlas (TCGA) [10] dataset and the Surveillance, Epidemiology, and End Results (SEER) [11] database which have been used extensively for cancer research.

However, consent, data ownership, privacy, intellectual property rights, and potential for misinterpretation of data [12] remain areas of concern to individuals who are more circumspect about changing the data sharing norm. To date, there has been no published synthesis of views on data sharing from the perspectives of diverse professional stakeholders. Thus, we conducted a systematic review of the literature on the views of researchers and healthcare professionals regarding the sharing of health data.

## Methods

This systematic literature review was part of a larger review of articles addressing data sharing, undertaken in accordance with the PRISMA statement for systematic reviews and meta-analysis [13]. The protocol was prospectively registered on PROSPERO (www.crd.york.ac.uk/PROSPERO, CRD42018110559).

The following databases were searched: EMBASE/MEDLINE, Cochrane Library, PubMed, CINAHL, Informit Health Collection, PROSPERO Database of Systematic Reviews, PsycINFO, and ProQuest. The final search was conducted on 21 October 2018. No date restrictions were placed on the search; key search terms are listed in Table 1. Papers were considered eligible if they: were published in English; were published in a peer review journal; reported original research, either qualitative or quantitative with any study design, related to data sharing in any disease setting; and included subjects over 18 years of age. Systematic literature reviews were included in the wider search but were not included in the results. Reference list and hand searching were undertaken to identify additional papers. Papers were considered ineligible if they focused on electronic health records, biobanking, or personal health records or were review articles, opinion pieces/articles/letters, editorials, or theses from masters or doctoral research. Duplicates were removed and title and abstract and full-text screening were undertaken using the Cochrane systematic literature review program Covidence [14]. Two authors were involved in all stages of the review process; conflicts were resolved by consensus.

**Table 1:** Key search criteria

(data sharing) OR (data link*) OR (secondary data analysis) OR (data reuse) OR (data mining)	
AND	
(real world data) OR (clinical trial) (medical record*) OR (patient record*) OR (routine data) OR (administrative data)	
AND	
attitud* OR view* OR opinion* OR perspective* OR satisfaction	
AND	
(breast cancer) OR (breast neoplasm) OR (breast tumo*) OR (Carcinoma, breast)	
AND/OR	
patient* OR consumer*	
AND/OR	
doctor* OR clinician OR oncologist OR specialist	
AND/OR	
Researcher* OR scientist* OR ‘data custodian’	

*Search includes ‘wildcards’ or truncation

Quality and bias were assessed at a study level using the QualSyst system for quantitative and qualitative studies as described by Kmet et al. [15]. A maximum score of 20 is assigned to articles of high quality and low bias; the final QualSyst score is a proportion of the total, with a possible score ranging from 0.0 to 1.0 [15].

Data extraction was undertaken using a pre-piloted form in Microsoft Office Excel. Data points included author, country and year of study, study design and methodology, health setting, and key themes and results. Where available, detailed information on research participants was extracted including age, sex, clinical/academic employment setting, publication and grant history, career stage, and world region.

Quantitative data were summarised using descriptive statistics. Synthesis of qualitative findings used a meta-ethnographic approach, in accordance with guidelines from Lockwood et al. [16].The main themes of each qualitative study were first identified and then combined, if relevant, into categories of commonality. Using a constant comparative approach, higher order themes and subthemes were developed. Quantitative data relevant to each theme were then incorporated. Using a framework analysis approach as described by Gale et al. [17], the perspectives of different professional groups (researchers, healthcare professionals, data custodians, and ethics committees) towards data sharing were identified. Where differences occurred, they are highlighted in the results. Similarly, where systematic differences according to other characteristics (such as age or years of experience), these are highlighted.

## Results

**Fig. 1:**
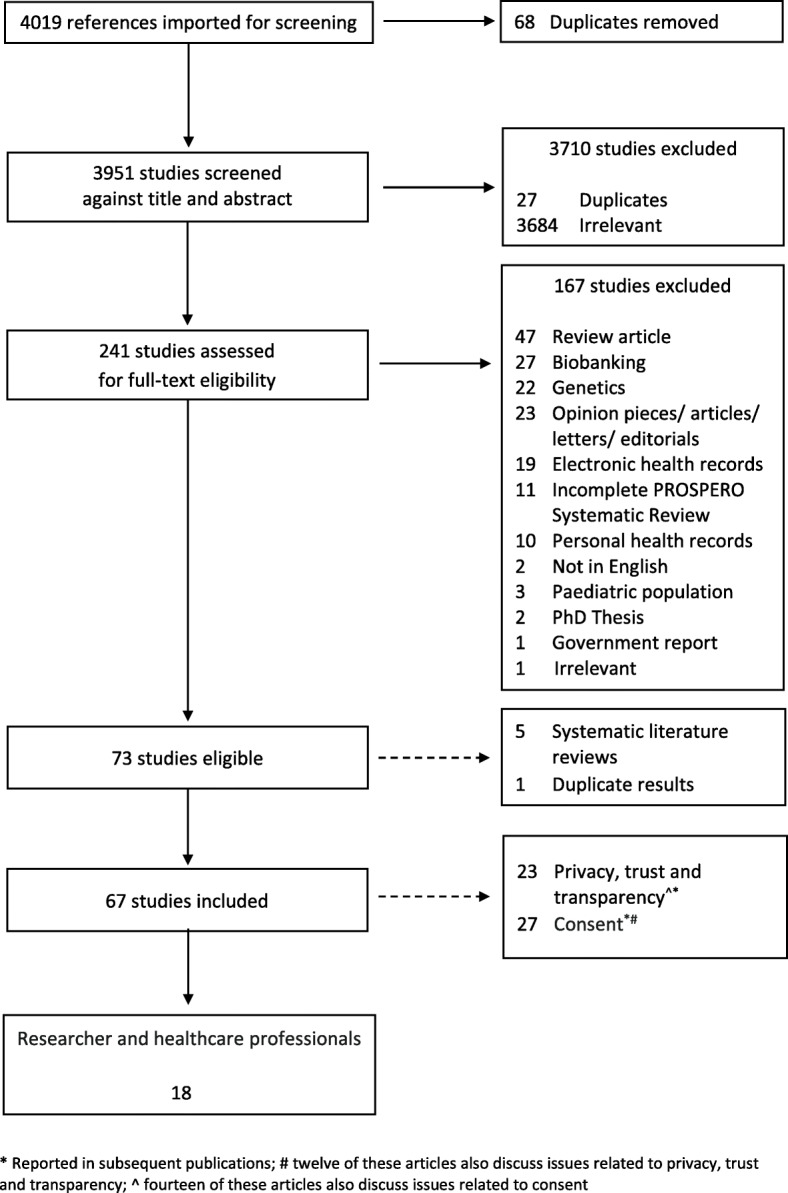
PRIMSA flow diagram (attached)

This search identified 4019 articles, of which 241 underwent full-text screening; 73 articles met the inclusion criteria for the larger review. Five systematic literature reviews were excluded as was one article which presented duplicate results; this left a total of 67 articles eligible for review. See Fig. 1 for the PRISMA diagram describing study screening.

This systematic literature review was originally developed to identify attitudes towards secondary use and sharing of health administrative and clinical trial data in breast cancer. However, as there was a paucity of material identified specifically related to this group, we present the multidisciplinary results of this search, and where possible highlight results specific to breast cancer, and cancer more generally. We believe that the material identified in this search is relevant and reflective of the wider attitudes towards data sharing within the scientific and medical communities and can be used to inform data sharing strategies in breast cancer.

Eighteen [18–35] of the 67 articles addressed the perspectives of clinical and scientific researchers, data custodians, and ethics committees and were analysed for this paper (Table 2). The majority (*n* = 16) of articles focused on the views of researchers and health professionals, [18–22, 24–26, 28–35], only one article focused on data custodians [27] and ethics committees [23] respectively. Four articles [18, 19, 21, 35] included a discussion on the attitudes of both researchers and healthcare professionals and patients; only results relating to researchers/clinicians are included in this analysis (Fig. 1).

**Table 2:** Included studies

Author, location, date of data collection	Methodology, sampling, analysis	Health condition/setting	No. of participants (***N***)	Participant demographics, ***n*** (%)	Key themes (alphabetically) relating to data	Outcomes, result(s)	QualSyst scores
**Qualitative studies**							
[18], Japan, November 2002	Focus group, thematic	General health	7	**Age**, **years** Between 37 and 44 ***Career Stage*** 12–17 years of experience **Discipline** 5 (72), internal medicine; 1 (14), emergency/intensive care; 1 (14), anaesthesiology **Sex** 7 (100), male **Other** Respondents involved in both clinical practice and research activities	Consent	***Consent*** Prior permission to access archived information/medical records was desirable. Individual informed consent complex to obtain in each case; patients provided opportunity to ‘opt out’ of research. Procedure for permission to use medical records varied between hospitals. Some researchers take for granted access to archived medical information without patients’ permission.	0.95
[19], England and Northern Ireland, February to July 2006	Interviews and focus groups, purposively sampled, constant comparative method	MS	68	***Discipline*** Neurologists, MS nurses, health service management professionals, researchers, representatives from pharmaceutical companies, social care professionals. ***Other*** 13, interviews; 10, focus groups	Access, systems, and metadata; consent; reasons for sharing; views on sharing	***Access***, ***systems***, ***and metadata*** No one individual or body should be responsible for the security and access to data. Stringent access controls required. Access should be monitored by a committee. • Direct access by pharmaceutical companies and marketing agencies was not considered appropriate. • Professionals more cautious than patients with MS using personal data within a register Prospective rights of patients must be protected to ensure privacy, including as a result of future developments. ***Consent*** Levels of involvement should be identified during consent process (i.e. from anonymised studies to direct participation). ***Reasons for sharing*** Facilitate short-term benefits: improved delivery of care, communication and receipt of information, quality of life. ***Views on sharing*** Altruistic attitudes towards the use of patient data in a register.	0.8
[20], South Africa, May to September 2014	Interviews and group interviews and two focus groups, thematic analysis	HIV/AIDS, TB	32	***Age***, ***years*** 39.3–48.6, average at sites ***Career Stage*** 10 (31), junior researchers; 4 (13), research managers; 10 (31), senior researchers; 3 (9), policy and department managers; 3 (9), executive members ***Discipline*** (***sites***) 1 (3), HIV/AIDS; 1 (3), fundamental research and specimen collection; 1 (3), social scientific research **Sex** 22 (69), female ***Other*** 20 (62), interviews and groups; 12 (38), focus groups	Access, systems, and metadata; consent; curation; experience of sharing; reasons for sharing; reasons for not sharing; views on sharing	***Access***, ***systems***, ***and metadata*** Proposed access to data varied from restricted/gate kept to requiring researchers to relinquish control over data collections once curated, or on release, unless embargoed. ***Consent*** The original consent must be respected in future research. Researchers must inform participants about data sharing plans (immediate and future); a broad approach to consent was recommended. ***Curation*** Data should be in a retriable and auditable format to ensure that the data is accurately preserved and not misused. After publication of primary analysis, there should be limited constraints or restrictions on the reuse of curated data. ***Experience of sharing*** Data sharing was either ad hoc/informal or through formal procedures enforced by institutional policy/contractual agreements. ***Reasons for data sharing*** Reasons include: move the field of science forward by opening new avenues of science or by closing knowledge gaps; collaborative communication; enhanced responsiveness of public health needs; validation of scientific outputs; reduced duplication of scientific effort; minimise research costs; an overwhelming public health interest or to minimise a disaster. ***Reasons for not sharing*** Competitive values of research data, including advancing researcher careers. Not all data is ‘equal’ and should only be shared in certain circumstances. ***Views on sharing*** No categorical objections to sharing de-identified data for academic and public health purposes, however there was disagreement about the extent to which research data should be shared beyond this. Extent of data sharing depends on the nature of the research question and whether the data could answer the question. Some respondents suggested that all data be shared, all of the time.	0.95
[21], Scotland, February and June 2011	Focus groups and interviews, thematic	General health	27	***Discipline*** 17 (63), GP; 10 (37), practice managers ***Respondents*** GP’s practice managers and health service researchers in two Scottish health boards	Access, systems, and metadata, curation, reasons for sharing, views on sharing	***Access***, ***systems***, ***and metadata*** Security and confidentiality concerns were expressed data. Assurances about the security, including accountability and transparency mechanisms were important. GPs may be able to block patient involvement by refusing access records or by not giving permission for the data extraction from their clinical system. Use of deceased patient data was a concern and required measures to prevent. ***Curation*** Concerns about impact on workload. ***Reasons for sharing*** Increased accessing to and recruitment patients for research/rapid access to a wider pool of patients. ***Views on sharing*** Clear support for the concept of a research register.	1
[22], Australia, January to October 2007	Interviews, thematic analysis	General health	20	***Career stage*** From registrar to ‘25 plus’ years ***Discipline*** GP ***Qualifications*** Overseas trained doctor awaiting Australian recognition to post graduate qualifications in Public Health, Obstetrics, Anaesthesiology, and Doctor of Philosophy. ***Sex*** 16 (55) males	Access, systems, and metadata; curation; policy framework; reasons for sharing; reasons for not sharing	***Access***, ***systems and metadata*** Resistance towards unspecified data amalgamating HI systems due to: perceptions or attitudes about unwanted functionality (do not want/need), inadequate attributes (capability and receptivity), or undesirable impact on the clinician’s role (autonomy, status, control and workflow). ***Curation*** Respondents did not want to impact on current workflow or allocated time addressing ‘nonmedical’ issues. Standardisation of processes and share clinical notes was noted. ***Policy framework*** Little to no interest in potential use of de-identified and delinked (not linked to other data such as demographic) amalgamated medical data. Use would be facilitated if it were shown to have positive consequences that were closely aligned to improved patient outcomes, improved GP workflow, a clear and certain potential to advantage, and streamlined interaction with outside entities. ***Reasons for sharing*** Regardless of context, all respondents identified potential benefits from being able to access consolidated longitudinal patient records, and to a lesser extent linked statistical data. ***Reasons for not sharing*** The potential for competitive disadvantage, the resolution of ethical, moral, and legal issues, the availability of appropriate technology, and motivations for sharing (political and policy).	0.9
[23], Canada, not provided	Interview, thematic	Ethics boards	30	***Employment setting*** 30 (47), university-based; 16 (53), hospital-based ***Discipline*** 6 of 16 were specialised ***Location*** 19 (63), Ontario or Quebec ***Other*** 2 (median) (range 1–6) people per interview	Consent	Note: Only data relating to the scenario involving retrospective medical record review are reported ***Consent*** *Requirement for* 47%, required individual patient consent; 10% would depend on how potentially identifying variables would be managed; 38% did not require consent; 7% suggested a notification and opt-out process. Most agreed that consent would be required if identifiable information was being extracted. Among those not requiring consent: substantial variation in recognising that the extracted information could potentially indirectly re-identify individuals. *Sites that required consent* (*n =* 14, 47*%*), *reasons for* 47% the principle of respect; 36% legislative requirements; 14%, had a general policy requiring consent in such circumstances; 21% if consent was feasible, it should be sought; 71% indicated that data allowed potential re-identification of individuals, and therefore consent was required (ethnicity, date of birth, and postcode of mother etc.); 47% concerned about external access to the billing or the health records; 50% external access to identifiable records was either the reason for requiring consent or an important factor. Several noted the fact that researchers would be going through the record itself, which, by nature, is identifying. *Sites that stated it depends* (*n =* 3, 10*%*), *reasons for* Whether or not consent would be required hinged entirely on the potential for indirectly identifying individuals from the combination of full postcode with ethnicity or date of birth. If this information was essential, then consent would be required. If not or truncated postcode or age category were used consent would not be required. No respondents were concerned about external access to records. *Sites not requiring* (*n =* 10, 38%), *reasons for* 70% minimal risk, nature of the research, as the rationale for not requiring consent. Deemed minimal risk because either: lack of direct contact with individuals or anonymity of the data being extracted from the health record. 40%, had policy is to not require consent for research involving retrospective chart review; 20%, indicated that their provincial body specifically permitted release of personal information without consent for research purposes if they believe that the researcher will protect the patient’s identity.	0.85
**Quantitative studies**							
[24], 13 African countries, August 2016	Survey, number and percentage of respondents	Life sciences	100	***Academic productivity***, ***articles*** Over last 5 years: 26, none; 42 (42), 1–3; 8 (8), 3–5; 24 (24), > 5 ***Career Stage*** 14 (14), professor; 57 (57), lecturer/researcher; 3 (3), post-doctoral researcher; 26 (26), postgraduate student ***Employment setting*** 60 (60), university; 27 (27), government research; 10 (10), independent research facility; 3 (3), industry ***Funding history*** 27 (27), international grant; 45 (45), national grant; 2 (2), private sector; 6 (6), internal funding; 20 (20), no funding ***Other*** Low-income countries ***Respondents*** Members of the NEPAD-SANBio network	Experience of sharing, promotion/professional criteria, reasons for sharing, reasons for not sharing	***Experience of sharing*** 60% happy to share data pre-publication with people that they knew, only 13% when asked to share with people that they did not know. 74% happy to share data post-publication with people they knew compared to 65% with people that they did not know. ***Promotion/professional criteria*** 17% strongly agreed that data sharing was not part of their promotion criteria; 31% agreed. ***Reasons for sharing*** 41% contributes to the advancement of science. 47% brings networking and collaboration opportunities. ***Reasons for not sharing*** 34% other researchers take their results. 29% having their data misinterpreted or misattributed. 23% missing out on opportunities to maximise intellectual property. 14% losing out on opportunities to maximise their publications.	0.95
[25], USA, Spring 1985	Survey, logic regression analysis	General sciences	790 57	***Employment setting*** 3, North American universities; non-science academics in public and private sectors. ***Other*** 790, researchers; 57, non-academic scientists	Experience of sharing; differences by age, discipline, professional focus, and world region; reasons for sharing; reasons for not sharing; research funding	***Experience of sharing*** 14% in sociology to 20% in chemistry (overall rate) refused to share raw data. When requested, and allowed by contractual agreements or employers, most professed to share. 87% of the time participants shared data. 59% claimed their colleagues were not prone to data sharing. ***Differences by age***, ***discipline***, ***professional focus and world region*** Few differences between fields of science or types of institution. ***Reasons for data sharing*** The principle of data sharing was a desirable norm. ***Reasons for not sharing*** Biotechnical fields cite financial (loosing patent rights, future grants or a reduction in publications) reasons. 72% in social sciences feared being pre-empted in the publication of findings. ***Research funding*** No statistically significant difference between sharing and the amount of past private or public financial support.	0.7
[26], international, 17 October 2013 to 19 March 2014	Survey, regression analysis	General science	595	***Career Stage*** 333 (56), researchers and analysts or senior academics; 101 (17), early career academics; 77 (13), students ***Discipline*** 119 (20), natural sciences; 119 (20), physical sciences; 71 (12), health, social and humanities; 54 (9), education, law and business ***Employment setting*** 446 (75), academics; 95 (16), government; 54 (9), commercial and non-profit agencies ***Location*** 262 (44), USA; 274 (46), ROW ***Respondents*** DATAOne Usability and Assessment Working Group	Access, systems, and metadata; experience of sharing; predictors of sharing and norms	***Access***, ***systems***, ***and metadata*** Reported use of metadata was predictive of data reuse. ***Experience of sharing*** Data reuse and data sharing were not linked, being only moderately correlated (r = 0.25). Self-reported data reuse behaviour, mean, 3.5 (SD 1) (scale of 0 to 5) (*n* = 589). *Data collection behaviour* ~ 50%, strongly favoured collecting data themselves; ~ 45%, from their team; ~ 30%, close colleagues; <10%, not appropriate to ask a librarian or data manager for (suitable) data. Sharing data compared to self-reported data reuse only moderately correlated; 10%, shared no data; 43%, shared some; 32%, most; 16%, all data. ***Predictors of data sharing and norms*** Efficacy and efficiency of data reuse predicts data reuse. Norms against data reuse predicts less reuse. Perception of the importance of data reuse predicts increased data reuse. Concerns about the trustworthiness of data did not predict less reuse of data. Remotely sensed data was strongly associated with increased reuse, as was reuse of metadata. Subjective norms about data reuse predict data reuse behaviour. Perceived norms, showed a large positive effect for the perceived importance of being able to reuse data. The non-effect of trust, respondents especially those with experience, are aware of the lack of acceptance of data reuse.	0.9
[27], international, 2010 and 2011	Survey, structured content analysis	All disciplines	24	***Discipline*** 4 (17), social sciences; 3 (13), humanities; 2 (8), interdisciplinary; 8 (33), biology; 7 (29), physical sciences, atmospheric sciences, geology, or astronomy ***Employment setting*** 8 (33), government; 7 (29), university; 9 (38), mixed ***Respondents*** Bench and physical sciences	Access, systems, and metadata; differences by age, discipline, professional focus and world region; reasons for not sharing	***Access***, ***systems***, ***and metadata*** 58.3%, ensuring attribution was the dominant reason for controlling use of data; 33.3%, restricting commercial research; 20.8%, protecting sensitive (non-personal) information.; 12.5%, limiting certain types of research; 8.3%, allowing depositors to decide; 8.3%, ensuring exclusivity; 12.5%, other (limiting to certain groups, protecting security of physical locations); 8.3%, not stated. Requirement to ‘report back to the repository or researcher regarding the use of data’ appeared in every discipline except humanities. ***Differences by age***, ***discipline***, ***professional focus and world region*** *Discipline*: More policy restrictions in biology repositories compared to social science repositories. ***Reasons for not sharing*** Reasons included avoiding data misuse; prohibition of further unapproved dissemination; prohibition on selling data; intellectual property concerns. Privacy concerns only noted in biology, humanities and social sciences.	0.9
[28], Germany, November to December 2013	Survey, deductive coding and multivariable analysis	All disciplines	603	***Age***, ***years*** 37 (average) ***Discipline*** 277 (46), economics; 235 (39), social sciences and sociology **Location** 458 (76), Germany; 145 (24), ROW ***Respondents*** Secondary data users and academic researchers ***Sex*** 368 (61), male	Experience of sharing; ownership; policy frameworks; promotion/professional criteria	***Experience of sharing*** Sharing process can be divided into six descriptive categories: data donor, research organisation, research community, norms, data infrastructure, and data recipients. ***Ownership*** Research data cannot be regarded as knowledge commons. ***Policy Framework*** Conceptual framework was developed to explain the process of data sharing from the primary researcher’s point of view. Research policies that better incentivise data sharing are needed to improve the quality of research results and foster scientific progress. ***Promotion/professional criteria*** Data sharing not a requirement of professional/academic promotion.	1
[29], USA, April to May 2014	Survey, odds ratio and Fisher exact test, worst case sensitivity testing	General science	135	***Discipline*** 113 (84), scientific; 22 (16), clinical ***Respondents*** Clinical and basic science researchers	Access, systems, and metadata; acknowledgement; curation; experience of sharing; reasons for sharing; reasons for not sharing; views on sharing	***Access***, ***systems***, ***and metadata*** 72% included some additional materials when they shared data; 47% shared contextualising information (metadata or a description of the experimental protocol). Sharing directly with other researchers was common, but most did not have experience uploading data to repositories. ***Acknowledgement*** (***n =*** **104**), ***after sharing data*** 31%, no publication had arisen; 51%, co-author on a publication; 35%, acknowledgement section of the publication; 22%, bibliography of the publication; 15%, not acknowledged. ***Curation***, ***time*** 28%, >10 hours; 29%, no additional time at all; 0%, data already existed in a shareable format. ***Experience of sharing*** Low levels of data sharing experience; relevance of re-using was higher than their expertise in doing so (same between clinical and scientific). 71%, shared directly with another researcher. 73%, scientific staff had shared data with another researcher 64%, clinical research staff had shared. 1.5-fold increased odds of sharing data in the scientific group (OR = 1.51, 95% CI: 0.577 to 3.955), this result is not statistically significant (*p* = 0.399). ***Reasons for sharing*** 69%, collaborate with other researchers; 64%, desire to advance science in a particular area; 49%, to assist a known colleague ***Reasons for not sharing*** (*n* = 20) Some researchers, particularly clinical staff, do not see sharing data in a repository as relevant to their work. 5 (100%) clinical compared to 2 (13%) of the scientific researchers indicated privacy was a concern. ***Repositories*** 27% and 24% rated uploading to data repositories as ‘very highly’ or ‘highly’ (%) relevant to their work respectively, but experience levels low. *Scientific staff* Relevance of sharing data in a repository more highly ranked than their expertise in doing so. More likely to consider sharing data in a repository relevant to their work. The odds of having HIGH relevance in the scientific group are 5.75 times larger than in the clinical group. The odds of having HIGH expertise in this task in the scientific group are also greater than in the clinical group. *Clinical staff* Relevance of sharing data in a repository more highly ranked than their expertise in doing so. 61%, never uploaded data to a repository. Scientific researchers regarded uploading data to a repository for sharing higher than those of the clinical researchers. ***Views on sharing*** 31%, rated the relevance of finding and re-using data as high, 29% rated it as very high. Odds of ranking data reuse as having high relevance in the scientific group were 4.26 times greater than the clinical group. In terms of expertise, the odds of having a high expertise ranks in the scientific group are also greater that the clinical staff. Therefore, compared to clinical researchers’ scientific researchers are more likely to consider data reuse highly relevant to their work.	1
[30], USA, 5 October 2015 to 30 November 2015	Survey, non-response analysis and ANOVA	General health	161	***Age***, ***years*** 4 (3), 25–34; 24 (15), 35–44; 39 (24), 45–54; 63 (39), 55–64; 28 (17), 65+; 3 (1), missing ***Career Stage*** 19 (12), assistant professor; 41 (26), associate professor; 74 (46), full professor; 7 (4), professor emeritus; 4 (3), lecturer/instructor; 2 (1), post-doctoral fellow; 5 (3), researcher; 3 (2), graduate student; 6 (4), other ***Discipline***, ***selected*** 14 (9), nursing; 11 (7), clinical medicine, other; 11 (7), oncology/cancer research ***Respondents*** Health scientists from the COS. ***Sex*** 54 (34) female ***Qualifications*** 1 (< 1), Bachelor’s degree; 12 (8), Master’s degree; 148 (92), PhD/doctoral degree	Experience of sharing; predictors of sharing and norms; reasons for sharing; reasons for not sharing	***Experience of sharing*** Measures were positively associated with data reuse intention among health scientists. The result shows that four exogenous latent variables including attitude, social norm, research climate, and organisational support, positively affect researchers’ intention to reuse data. ***Predictors of sharing and norms*** The effects of social norm (*β* .0.339; *p* < 0.01) and attitude (*β* .0.331; *p* < 0.01) were relatively higher than other factors. Perceived usefulness and perceived concern were found to have indirect effects on intention of data reuse through attitude. A positive social norm towards data reuse positively supports researchers’ data reuse intention. ***Reasons for sharing*** The perceived usefulness is found to be the strongest indicator that is indirectly influential to reuse intention. ***Reasons for not sharing*** Negative association with data reuse practice among health scientists. Legal issues relating to privacy, cultural barriers, and technical challenge were cited. Must comply with laws, regulations, and protocols prescribing how to securely manage information. Legal uncertainty. Managing information when sharing clinical trials’ research data. Scientific competition and lack of incentives. The quality of secondary data use still remains to be addressed.	1
[31], international, July 2012 to September	Survey, characteristics of survey respondents: non-respondents, we used χ2 tests for categorical variables and the Kruskal-Wallis test for continuous variables (trial enrolment), using two-sided tests with a type I error level of 0.05.	General health	317	***Academic productivity***, ***articles*** Last 3 years: 71 (22), 1–10; 117 (37), 11–25; 129 (41), ≥ 25 ***Age*** 126 (40), ≤ 49; 159 (50), 50–64; 31 (10), ≥ 65 ***Career stage*** Completed training 10–24 years ago; two thirds of these had reached; the rank of full professor. ***Employment setting*** 278 (88), medical school or hospital; 19 (6), government; 4 (1), private industry; 16 (5), other ***Funding history*** 46% had been awarded 4 or more grants; 52% had received > $1million in direct research support ***Qualifications*** Training in the USA or Canada ***Sex*** 73 (23), female	Experience of sharing; reasons for sharing; reasons for not sharing; views on sharing	***Experience of sharing*** 74% sharing de-identified data through data repositories should be required 72% believed investigators should be required to share de-identified data in response to individual requests. 18% were required by trial funder to deposit the trial data in a repository; of which 57% had done so. 47% had received an individual request to share their clinical trial data; of these, 77% had granted and 38% had denied at least one request. ***Reasons for sharing data*** 88% of respondents supported data sharing; 78% promoting open science; 42% academic benefits and recognition. ***Reasons for not sharing data*** 65% concerned about (in)appropriate data use; 41% investigator and funder interests; 29% protection of research subjects ***Views on sharing*** *Right of first use of clinical trial data* 2% data should be made available to investigators external to the study team immediately on trial completion; 34% within 1 to 2 years of trial completion; 31% within 3 to 5 or more years of trial completion; 33% no time limit and that the right of first use should extend until the main findings are accepted for publication.	1
[32], international, July 2012 to early September	Survey, secondary analysis	General health	317	***Academic productivity***, ***articles*** Last 3 years: 71(22), 1 to 10; 117 (37), 11 to 25; 129 (41), > 25 ***Age***, ***years*** 126 (40), ≤ 49; 159 (50), 50 to 64; 31 (10), ≥ 65 ***Funding history*** 120 (38)—government; 152 (48)—mixed ***Location*** 167 (53), US or Canada; 113 (36), Western Europe; 37 (11), other ***Sex*** 243 (77), male	Experience of sharing; reasons for sharing; reasons for not sharing; trust	***Experience of sharing*** No significant differences in support for data sharing in principle between respondents by trialists’ academic productivity and geographic location, trial funding source and size, and the journal in which it was published. Rates of support between 81% and 100%. No significant differences in reasons for withholding data between respondents categorised trialists’ academic productivity and geographic location, trial funding source and size, and the journal in which it was published. • Academically productive respondents (>25 articles published over the past 3 years) responded affirmatively least frequently (24%), as compared to respondents who published 1 to 10 articles (41%), and 11 to 25 articles (40%). • Respondents who received industry funding also responded affirmatively least frequently (24%), as compared to respondents who received government funding (42%), and non-profit funding (44%). ***Reasons for sharing*** 78%, promotion of open science. No significant differences in reasons for sharing data between respondents by trialists’ academic productivity and geographic location, trial funding source and size, and the journal in which it was published. An exception to this was, has or would share data from their published study in order to receive academic benefits or recognition based on geographic location (*p* < 0.001). Western Europe responded affirmatively 58% compared to 31% in the US or Canada, and 43% ROW. ***Reasons for not sharing*** Rates of overall concern ranged between 67 and 84%. No significant differences in overall concern about sharing data through repositories between respondents by trialists’ academic productivity and geographic location, trial funding source and size, and the journal in which it was published. 74% identified ensuring appropriate data use (65%) as a reason for withholding data from their published study. Concerns included data not appropriate for the requested purpose, and the potential for misinterpretation and misleading secondary analyses. Prevention of misleading secondary analyses and misinterpretation of data. ***Trust*** Mistrust of the data requester’s intent.	1
[33], international, 27 October 2009 to 31July 2010	Survey, not described	General sciences	1329	***Age***, ***years*** 453 (38), 20–39; 359 (30), 40–50; 393 (33), > 50; mean: 44.8 ***Career Stage*** 137 (10.5), assistant professor; 187 (14.3), associate professor; 291 (22.2), professor; 276 (21.1), researcher; 177 (13.5), student **Discipline** 475 (36.1), environmental sciences and ecology; 204 (15.5), social sciences; 181 (13.7), biology; 158 (12.0), physical; 118 (9.0), sciences; computer science/engineering; 98 (7.4), other; 52 (3.9), atmospheric science; 31 (2.4), medicine ***Employment setting*** 1058 (80.5), academic; 167 (12.7), government; 34 (2.6), commercial; 35 (2.7), non-profit; 21 (1.6), other **Location** 930 (73), N. America; 188 (15), Europe; 94 (7.3) Asia/Oceania ***Sex*** Two thirds, male	Access, systems, and metadata; acknowledgement; curation; differences by age, discipline, professional focus and world region; experience of sharing; reasons for sharing; reasons for not sharing; views on sharing	NB: see also [34] for follow up results ***Access***, ***systems***, ***and metadata*** 43% have the sole responsibility for all their datasets; 37% have for some of their datasets, and 21% do not. 56% did not use any metadata standard; ~ 22% used their own metadata standard. *Central repository with all data and no restrictions*: 41% to 52% respondents in most disciplines agree with this statement, with medicine (17%) and social sciences (32%) even less likely to agree. ***Acknowledgement*** 92%, important that their data are cited when used by other researchers; 86%, appropriate to create new datasets from shared data; 52%, fair to disseminate results based (at least in part) on data without the data provider’s approval. 69% indicated that paying for the costs of data does not include the right to use that data or that they do not believe that data users should be required to pay data creators. ***Curation*** 59.8% (agree strongly or somewhat) they are satisfied with cataloguing or describing their data. 45% and 73% are satisfied with the process of storing data beyond the life of the project compared to short term, respectively. 35% of the respondents stated that they are dissatisfied with the long-term storage process. 46%, do not make their data electronically available to others. <6% of scientists who make ‘all’ of their data available via some mechanism, tends to re-enforce the lack of data sharing within the communities surveyed. ***Differences by age***, ***discipline***, ***professional focus and world region*** Not all scientists share data equally or have the same perceptions of data sharing and reuse. *Age* 40–50 years: less likely to agree, than other age groups, that their organisations have processes for managing data sharing during and after the project. Younger: less likely to agree to share all data without restrictions, but more likely to agree to share some as long as restrictions are in place. Younger more likely to think lack of access to data is a major impediment to progress in science and has restricted their ability to answer scientific questions. *Discipline* Majority shared data with others, but respondents from medical fields and social sciences were less likely to make their data electronically available. *Professional focus* 74% and 79% of research-intensive respondents and teaching-intensive respondents showed willingness to place some data into a central data repository with no restrictions, and willingness to share across broad group of researchers who use data in different ways, 77% and 83% respectively. *World region* Non-N. America/non-European’s more likely to think that lack of access to data is a major impediment to progress in science (Other = 79%, Europe =72%, and N. America =64%) and has restricted their ability to answer scientific questions (Other = 63%, Europe = 55%, and N. America 47%). ‘Other’ parts of the world are most willing to place all of their data into a central data repository with no restrictions (53%); more likely to make their data available if they could place conditions on access (73%); and the most satisfied with their ability to integrate data from disparate sources to address research questions (58%). ***Experience of sharing*** Nearly one third of the respondents chose not to answer whether they make their data available to others. ***Reasons for sharing*** ~ 60% agree that lack of access to data generated by other researchers or institutions is a major impediment to progress in science. ***Reasons for not sharing*** Reasons deeply rooted in the practices and culture of the research process as well as the researchers themselves. 53.6%, insufficient time. 39.6%, lack of funding. 24.1%, do not have rights to make public. 264 (23.5%), no place to store. 19.8%, lack of standards. 17.4%, sponsor does not require. 15.0%, do not need. 14.6%, other. 14.4%, should not be available. 75%, data may be misinterpreted due to complexity of the data across their research field. 71% data may be misinterpreted due to poor quality of data across their research field. 74%, data may be used in other ways than intended across their research field. 67% agreed that lack of access to data generated by other researchers or institutions is a major impediment to progress in science. 50% reported that lack of access to data generated by other researcher or institution has restricted their ability to answer scientific questions. ***Views on sharing*** 43%, organisation or project had a formal process for managing data during the life of the project. 47%, disagreed that their organisation or project has a formal. established process for storing data beyond the life of the project. 36%, agree that others can access their data easily. 14%, data ‘Should not be Available’.	0.8
[34], international, October 2009 to July 2010 and October 2013 to March 2014	Survey, ANOVA, chi-square tests	General sciences	1015	***Age***, ***years*** 380 (40.9), 20–39; 196 (21.1), 40–49; 352 (37.9), 50+ **Discipline**, **selected** 70 (7.1), biology; 12 (1.2), humanities; 37 (3.8) medicine/health science; 47 (4.8), physical sciences; 21 (2.1), psychology; 44 (4.5) social sciences ***Location*** 592 (61.0), N. America; 91 (9.4), Asia; 141 (14.5), Europe; 72 (7.4), Africa; 55 (5.7), S. America; 20 (2.1%), ANZ	Access, systems, and metadata; acknowledgement; differences by age, discipline, professional focus, and world region; reasons for not sharing; views on sharing	Note: Change between surveys reported: see also [33] **Access**, **systems**, **and metadata** Younger respondents had more restrictions on access to their data and agreed significantly less than older respondents that their data is easy to access. ***Acknowledgement*** An ongoing issue, and one likely to accompany the gradual institutionalization of emerging scientific practices over time. ***Differences by age***, ***discipline***, ***professional focus***, ***and world region*** *Age* Younger respondents more favourable towards data sharing and reuse yet make less of their data available than older respondents. Younger prioritise control over and credit for their work more than older researchers. Those ≥ 50 years claim to share significantly more than both the 40–49 years and 22–39 years age groups. *Discipline* Medicine/health sciences and others who work with human subjects were significantly less willing to share their data than other disciplines. No significant differences across subject disciplines when it came to perceived risks associated with data sharing. *World region* Asia: more strongly about data access as an important part of their own scientific pursuits; however, agreed more strongly than those from other geographic regions that permission was needed to access data. N. American: more wary of possible misuse of shared data. Were also less likely than Asian respondents to agree that conditions for use of their data were fair. ***Views on sharing*** Increased acceptance of and willingness to engage in data sharing. More agreement and willingness among scientists to share at least some or all of their data across broader groups with no limitations. Education and medicine/health science were more inclined to agree that they do not have the right to make their data available in the first place. ***Reasons for not sharing*** Increased perceived risk associated with data sharing. Misuse of shared data. Education, medicine/health science, and psychology more inclined than others to agree that their data should not be available for others to use in the first place.	0.85
**Mixed methods**							
[35], USA, date not reported.	Survey, qualitative and qualitative analysis	Mental health	8	***Discipline*** 3 (38), clinician; 3 (38), therapist; 1 (13), treatment coordinator; 1 (13), PhD nursing practice	Access, systems, and metadata; consent	***Access***, ***systems***, ***and metadata*** 87.5%, patients should have more choices for controlling access data. 75%, care could be negatively affected when patients restrict access to relevant clinical information. 25%, patient choice should expand. 25%, educating patients and providers about the positive and negative aspects of granular control. ***Consent*** Broad and does not reflect patient choices. Time required to implement consent and educate patients is a potential barrier to implementing a granular consent process. 37.5%, ‘time’ as the most significant barrier in implementing a system that permits more granular control of protected health information.	0.9

*ANZ* Australia and New Zealand, *AIDS* acquired immune deficiency syndrome, *CI* confidence interval, *COS* Community of Science Scholars, *GP* general practitioner, *HC* health care professionals, *HI* health information, *HIV* human immunodeficiency virus, *MS* multiple sclerosis, *NEPAD-SANBio* New Partnership for Africa’s Development—Southern Network for the Biosciences, *NIH* National Institutes of Health, *N*. north, *OR* odds ratio, *PHI* protected health information, *ROW* rest of world, *SD* standard deviation, *S*. south, *TB* tuberculosis, *USA* United States of America

### Study design, location, and disciplines

Several study methodologies were used, including surveys (*n* = 11) [24–27, 29–35], interviews and focus groups (*n* = 6) [18–23], and mixed methods (*n* = 1) [28]. Studies were conducted in a several countries and regions; a breakdown by country and study is available in Table 3.

**Table 3:** Studies by country

Country study undertaken (in alphabetical order)	Reference
Australia	[22]
Canada	[23]
England and Northern Ireland	[19]
Germany	[28]
Japan	[18]
Multiple	[26, 27, 31–34]
Scotland	[21]
Sub-Saharan Africa	[20, 24]
United States of America (USA)	[25, 29, 30, 35]

In addition to papers focusing on general health and sciences [18, 21, 22, 24–26, 29–34], two articles included views from both science and non-science disciplines [27, 28]. Multiple sclerosis (MS) [19], mental health [35], and human immunodeficiency virus (HIV)/acquired immunodeficiency syndrome (AIDS)/tuberculosis (TB) [20] were each the subject of one article.

#### Study quality

Results of the quality assessment are provided in Table 2. QualSyst [15] scores ranged from 0.7 to 1.0 (possible range 0.0 to 1.0). While none were blinded studies, most provided clear information on respondent selection, data analysis methods, and justifiable study design and methodology.

### Themes

**Fig. 2:**
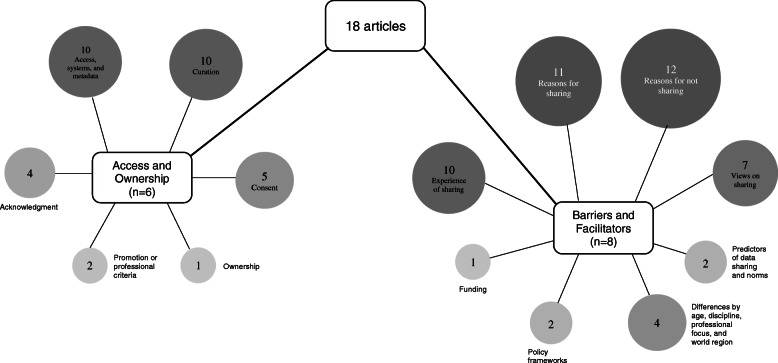
Graphic representation of key themes and subthemes identified (attached)

Four key themes, barriers, facilitators, access, and ownership were identified; 14 subthemes were identified. A graphical representation of article themes is presented in Fig. 2. Two articles reflect the perspective of research ethics committees [23] and data custodians [27]; concerns noted by these groups are similar to those highlighted by researchers and healthcare professionals.

### Barriers and facilitators

#### Reasons for not sharing

Eleven articles identified barriers to data sharing [20, 22, 24, 25, 27, 29–34]. Concerns cited by respondents included other researchers taking their results [24, 25], having data misinterpreted or misattributed [24, 27, 31, 32], loss of opportunities to maximise intellectual property [24, 25, 27], and loss of publication opportunities [24, 25] or funding [25]. Results of a qualitative study showed respondents emphasised the competitive value of research data and its capacity to advance an individual’s career [20] and the potential for competitive disadvantage with data sharing [22]. Systematic issues related to increased data sharing were noted in several articles where it was suggested the barriers are ‘deeply rooted in the practices and culture of the research process as well as the researchers themselves’ [33] (p. 1), and that scientific competition and a lack of incentive in academia to share data remain barriers to increased sharing [30].

Insufficient time, lack of funding, limited storage infrastructure, and lack of procedural standards were also noted as barriers [33]. Quantitative results demonstrated that the researchers did not have the right to make the data public or that there was no requirement to share by the study sponsor [33]. Maintaining the balance between investigator and funder interests and the protection of research subjects [31] were also cited as barriers. Concerns about privacy were noted in four articles [25, 27, 29, 30]; one study indicated that clinical researchers were significantly more concerned with issues of privacy compared to scientific researchers [25]. The results of one qualitative study indicated that clinicians were more cautious than patients regarding the inclusion of personal information in a disease specific registry; the authors suggest this may be a result of potential for legal challenges in the setting of a lack of explicit consent and consistent guidelines [19]. Researchers, particularly clinical staff, indicated that they did not see sharing data in a repository as relevant to their work [29]

Trust was also identified as a barrier to greater data sharing [32]. Rathi et al. identified that researchers were likely to withhold data if they mistrusted the intent of the researcher requesting the information [32]. Ethical, moral, and legal issues were other potential barriers cited [19, 22]. In one quantitative study, 74% of respondents (*N* = 317) indicated that ensuring appropriate data use was a concern; other concerns included data not being appropriate for the requested purpose [32]. Concerns about data quality were also cited as a barrier to data reuse; some respondents suggested that there was a perceived negative association of data reuse among health scientists [30].

#### Reasons for sharing

Eleven articles [19–22, 24, 25, 29–33] discussed the reasons identified by researchers and healthcare professionals for sharing health data; broadly the principle of data sharing was seen as a desirable norm [25, 31]. Cited benefits included improvements to the delivery of care, communication and receipt of information, impacts on care and quality of life [19], contributing to the advancement of science [20, 24, 29], validating scientific outputs, reducing duplication of scientific effort and minimising research costs [20], and promoting open science [31, 32]. Professional reasons for sharing data included academic benefit and recognition, networking and collaborative opportunities [20, 24, 29, 31], and contributing to the visibility of their research [24]. Several articles noted the potential of shared data for enabling faster access to a wider pool of patients [21] for research, improved access to population data for longitudinal studies [22], and increased responsiveness to public health needs [20]. In one study, a small percentage of respondents indicated that there were no benefits from sharing their data [24].

Analysis of quantitative survey data indicated that the perceived usefulness of data was most strongly associated with reuse intention [30]. The lack of access to data generated by other researchers or institutions was seen as a major impediment to progress in science [33]. In a second study, quantitative data showed no significant differences in reasons for sharing by clinical trialists’ academic productivity, geographic location, trial funding source or size, or the journal in which the results were published [32]. Attitudes towards sharing in order to receive academic benefits or recognition differed significantly based on the respondent’s geographic location; those from Western Europe were more willing to share compared to respondents in the USA or Canada, and the rest of the world [32].

#### Views on sharing

Seven articles [19–21, 29, 31, 33, 34] discussed researchers’ and healthcare professionals’ views relating to sharing data, with a broad range of views noted. Two articles, both qualitative, discussed the role of national registries [21], and data repositories [31]. Generally, there was clear support for national research registers and an acceptance for their rationale [21], and some respondents believed that sharing de-identified data through data repositories should be required and that when requested, investigators should share data [31]. Sharing de-identified data for reasons beyond academic and public health benefit were cited as a concern [20]. Two quantitative studies noted a proportion of researchers who believed that data should not be made available [33, 34]. Researchers also expressed differences in how shared data should be managed; the requirement for data to be ‘gate-kept’ was preferred by some, while others were happy to relinquish control of their data once curated or on release [20]. Quantitative results indicated that scientists were significantly more likely to rank data reuse as highly relevant to their work than clinicians [29], but not all scientists shared data equally or had the same views about data sharing or reuse [33]. Some respondents argued that not all data were equal and therefore should only be shared in certain circumstances. This was in direct contrast to other respondents who suggested that all data should be shared, all of the time [20].

#### Differences by age, background, discipline, professional focus, and world region

Differences in attitudes towards shared data were noted by age, professional focus, and world region [25, 27, 33, 34]. Younger researchers, aged between 20–39 and 40–49 years, were less likely to share their data with others (39% and 38% respectively) compared to other age groups; respondents aged over 50 years of age were more willing (46%) to share [33]. Interestingly, while less willing to share, younger researchers also believed that the lack of access to data was a major impediment to science and their research [33]. Where younger researchers were able to place conditions on access to their data, rates of willingness to share were increased [33].

Respondents from the disciplines of education, medicine/health science, and psychology were more inclined than others to agree that their data should not be available for others to use in the first place [34]. However, results from one study indicated that researchers from the medical field and social sciences were less likely to share compared to other disciplines [33]. For example, results of a quantitative study showed that compared to biologists, who reported sharing 85% of their data, medical and social sciences reported sharing their data 65% and 58% percent of the time, respectively [33].

One of the primary reasons for controlling access to data, identified in a study of data custodians, was due to a desire to avoid data misuse; this was cited as a factor for all surveyed data repositories except those of an interdisciplinary nature [27]. Limiting access to certain types of research and ensuring attribution were not listed as a concern for sociology, humanities or interdisciplinary data collections [27]. Issues pertaining to privacy and sensitive data were only cited as concerns for data collections related to humanities, social sciences, and biology, ecology, and chemistry; concerns regarding intellectual property were also noted [27]. The disciplines of biology, ecology, and chemistry and social sciences had the most policy restrictions on the use of data held in their repositories [27].

Differences in data sharing practices were also noted by world region. Respondents not from North American and European countries were more willing to place their data on a central repository; however, they were also more likely to place conditions on the reuse of their data [33, 34].

#### Experience of data sharing

The experience of data sharing among researchers was discussed in nine articles [20, 24–26, 28–33]. Data sharing arrangements were highly individual and ranged from ad hoc and informal processes to formal procedures enforced by institutional policies in the form of contractual agreements, with respondents indicating data sharing behaviour ranging from sharing no data to sharing all data [20, 26, 31]. Quantitative data from one study showed that researchers were more inclined to share data prior to publication with people that they knew compared to those they did not; post publication, these figures were similar between groups [24]. While many researchers were prepared to share data, results of a survey identified a preference of researchers to collect data themselves, followed by their team, or by close colleagues [26].

Differences in the stated rate of data sharing compared to the actual rate of sharing [25] were noted. In a large quantitative study (*N* = 1329), nearly one third of respondents chose not to answer whether they make their data available to others; of those who responded to the question, 46% reported they do not make their data electronically available to others [33]. By discipline, differences in the rate of refusal to share were higher in chemistry compared to non-science disciplines such as sociology [25]. Respondents who were more academically productive (> 25 articles over the past 3 years) reported that they have or would withhold data to protect research subjects less frequently than those who were less academically productive or received industry funding [32].

Attitudes to sharing de-identified data via data repositories was discussed in two articles [29, 31]. A majority of respondents in one study indicated that de-identified data should be shared via a repository and that it should be shared when requested. A lack of experience in uploading data to repositories was noted as a barrier [29]. When data was shared, most researchers included additional materials to support their data including materials such as metadata or a protocol description [29].

Two articles [28, 30] focused on processes and variables associated with sharing. Factors such as norms, data infrastructure/organisational support, and research communities were identified as important factors in a researcher’s attitude towards data sharing [28, 30]. A moderate correlation between data reuse and data sharing suggest that these two variables are not linked. Furthermore, sharing data compared to self-reported data reuse were also only moderately associated (Pearson’s correlation of 0.25 (*p* ≤ 0.001)) [26].

#### Predictors of data sharing and norms

Two articles [26, 30] discussed the role of social norms and an individual’s willingness to share health data. Perceived efficacy and efficiency of data reuse were strong predictors of data sharing [26] and the development of a ‘positive social norm towards data sharing support(s)[ed] researcher data reuse intention’ [30] (p. 400).

#### Policy framework

The establishment of clear policies and procedures to support data sharing was highlighted in two articles [22, 28]. The presence of ambiguous data sharing policies was noted as a major limitation, particularly in primary care and the increased adoption of health informatics systems [22]. Policies that support an efficient exchange system allowing for the maximum amount of data sharing are preferred and may include incentives such as formal recognition and financial reimbursement; a framework for this is proposed in Fecher et al. [28].

#### Research funding

The requirement to share data funded by public monies was discussed in one article [25]. Some cases were reported of researchers refusing to share data funded by tax-payer funds; reasons for refusal included a potential reduction in future funding or publishing opportunities [25].

### Access and ownership

Articles relating to access and ownership were grouped together and seven subthemes were identified.

#### Access, information systems, and metadata

Ten articles [19–22, 26, 27, 29, 33–35] discussed the themes of access, information systems, and the use of metadata. Ensuring privacy protections in a prospective manner was seen as important for data held in registries [19]. In the setting of mental health, researchers indicated that patients should have more choices for controlling access to shared registry data [35]. The use of guardianship committees [19] or gate-keepers [20] was seen as important in ensuring the security and access to data held in registries by some respondents; however, many suggested that a researcher should relinquish control of the data collection once curated or released, unless embargoed [20]. Reasons for maintaining control over registry data included ensuring attribution, restricting commercial research, protecting sensitive (non-personal) information, and limiting certain types of research [27]. Concerns about security and confidentiality were noted as important and assurances about these needed to be provided; accountability and transparency mechanisms also need to be included [21]. Many respondents believed that access to the registry data by pharmaceutical companies and marketing agencies was not considered appropriate [19].

Respondents to a survey from medicine and social sciences were less likely to agree to have all data included on a central repository with no restrictions [33]; notably, this was also reflected in the results of qualitative research which indicated that health professionals were more cautious than patients about the inclusion of personal data within a disease specific register [19].

While many researchers stated that they commonly shared data directly with other researchers, most did not have experience with uploading data to repositories [29]. Results from a survey indicated that younger respondents have more data access restrictions and thought that their data is easier to access significantly more than older respondents [34]. In the primary care setting, concerns were noted about the potential for practitioners to block patient involvement in a registry by refusing access to a patient’s personal data or by not giving permission for the data to be extracted from their clinical system [21]. There was also resistance in primary care towards health data amalgamation undertaken for an unspecified purpose [22]; respondents were not in favour of systems which included unwanted functionality (do not want/need), inadequate attributes (capability and receptivity) of the practice, or undesirable impact on the role of the general practitioner (autonomy, status, control, and workflow) [22].

Access to ‘comprehensive metadata (is needed) to support the correct interpretation of the data’ [26] (p. 4) at a later stage. When additional materials were shared, most researchers shared contextualising information or a description of the experimental protocol [29]. The use of metadata standards was not universal with some respondents using their own [33].

#### Curation

Several articles highlighted the impact of data curation on researchers’ time [20–22, 29, 33] or finances [24, 28, 29, 33, 34]; these were seen as potential barriers to increased registry adoption [21]. Tasks required for curation included preparing data for dissemination in a usable format and uploading data to repositories. The importance of ensuring that the data is accurately preserved for future reuse was highlighted; it must be presented in a retriable and auditable manner [20]. The amount of time required to curate data ranged from ‘no additional time’ to ‘greater than ten hours’ [29]. In one study, no clinical respondent had their data in a sharable format [29]. In the primary care setting, health information systems which promote sharing were not seen as being beneficial if they required standardisation of processes and/or sharing of clinical notes [22]. Further, spending time on non-medical issues in a time poor environment [22] was identified as a barrier. Six articles described the provision of funding or technical support to ensure data storage, maintenance, and the ability to provide access to data when requested. All noted a lack of funding and time as a barrier to increased sharing data [20, 24, 28, 29, 33, 34].

#### Consent

Results of qualitative research indicated a range of views regarding consent mechanisms for future data use [18–20, 23, 35]. Consenting for future research can be complex given that the exact nature of the study will be unknown, and therefore some respondents suggested that a broad statement on future data uses be included [19, 20] during the consent process. In contrast, other participants indicated that the current consent processes were too broad and do not reflect patient preferences sufficiently [35]. The importance of respecting the original consent in all future research was noted [20]. It was suggested that seeking additional consent for future data use may discourage participation in the original study [20]. Differences in views regarding the provision of detailed information about sharing individual level data was noted suggesting that the researchers wanted to exert some control over data they had collected [20]. An opt-out consent process was considered appropriate in some situations [18] but not all; some respondents suggested that consent to use a patient’s medical records was not required [18]. There was support by some researchers to provide patients with the option to ‘opt-in’ to different levels of involvement in a registry setting [19]. Providing patients more granular choices when controlling access to their medical data [35] was seen as important.

The attitudes of ethics and review boards (*N* = 30) towards the use of medical records for research was discussed in one article [23]. While 38% indicated that no further consent would be required, 47% required participant consent, and 10% said that the requirement for consent would depend on how the potentially identifying variables would be managed [23]. External researcher access to medical record data was associated with a requirement for consent [23].

#### Acknowledgement

The importance of establishing mechanisms which acknowledge the use of shared data were discussed in four articles [27, 29, 33, 34]. A significant proportion of respondents to a survey believed it was fair to use other researchers’ data if they acknowledged the originator and the funding body in all disseminated work or as a formal citation in published works [33]. Other mechanisms for acknowledging the data originator included opportunities to collaborate on the project, reciprocal data sharing agreements, allowing the originator to review or comment on results, but not approve derivative works, or the provision of a list of products making use of the data and co-authorship [33, 34]. In the setting of controlled data collections, survey results indicated that ensuring attribution was a motivator for controlled access [27]. Over half of respondents in one survey believed it was fair to disseminate results based either in whole or part without the data provider’s approval [33]. No significant differences in mechanisms for acknowledgement were noted between clinical and scientific participants; mechanisms included co-authorship, recognition in the acknowledgement section of publications, and citation in the bibliography [29]. No consentient method for acknowledging shared data reuse was identified [29].

#### Ownership

Data ownership was identified as a potential barrier to increased data sharing in academic research [28]. In the setting of control of data collections, survey respondents indicated that they wanted to maintain some control over the dataset, which is suggestive of researchers having a perceived ownership of their research data [28]. Examples of researchers extending ownership over their data include the right to publish first and the control of access to datasets [28]. Fecher et al. noted that the idea of data ownership by the researcher is not a position always supported legally; ‘the ownership and rights of use, privacy, contractual consent and copyright’ are subsumed [28] (p. 15). Rather data sharing is restricted by privacy law, which is applied to datasets containing data from individuals. The legal uncertainty about data ownership and the complexity of law can deter data sharing [28].

#### Promotion/professional criteria

The role of data sharing and its relation to promotion and professional criteria were discussed in two articles [24, 28]. The requirement to share data is rarely a promotion or professional criterion, rather the systems are based on grants and publication history [24, 28]. One study noted that while the traditional link between publication history and promotion remains, it is ‘likely that funders will continue to get sub-optimal returns on their investments, and that data will continue to be inefficiently utilised and disseminated’ [24] (p. 49).

## Discussion

This systematic literature review highlights the ongoing complexity associated with increasing data sharing across the sciences. No additional literature meeting the inclusion criteria were identified in the period between the data search and the submission of this manuscript. Data gaps identified include a paucity of information specifically related to the attitudes of breast cancer researchers and health professionals towards the secondary use and sharing of health administrative and clinical trial data.

While the majority of respondents believed the principles of data sharing were sound, significant barriers remain: issues of consent, privacy, information security, and ownership were key themes throughout the literature. Data ownership and acknowledgement, trust, and policy frameworks influenced sharing practice, as did age, discipline, professional focus, and world region.

Addressing concerns of privacy, trust, and information security in a technologically changing and challenging landscape is complex. Ensuring the balance between privacy and sharing data for the greater good will require the formation of policy and procedures, which promote both these ideals.

Establishing clear consent mechanisms would provide greater clarity for all parties involved in the data sharing debate. Ensuring that appropriate consent for future research, including secondary data analysis and sharing and linking of datasets, is gained at the point of data collection, would continue to promote research transparency and provide healthcare professionals and researchers with knowledge that an individual is aware that their data may be used for other research purposes. The establishment of policy which supports and promotes the secondary use of data and data sharing will assist in the normalisation of this type of health research. With the increased promotion of data sharing and secondary data analysis as an established tool in health research, over time barriers to its use, including perceptions of ownership and concerns regarding privacy and consent, will decrease.

The importance of establishing clear and formal processes associated with acknowledging the use of shared data has been underscored in the results presented. Initiatives such as the Bioresource Research Impact Factor/Framework (BRIF) [36] and the Citation of BioResources in journal Articles (CoBRA) [37] have sought to formalise the process. However, increased academic recognition of sharing data for secondary analysis requires further development and the allocation of funding to ensure that collected data is in a usable, searchable, and retrievable format. Further, there needs to be a shift away from the traditional criteria of academic promotion, which includes research outputs, to one which is inclusive of a researcher’s data sharing history and the availability of their research dataset for secondary analysis.

The capacity to identify and use already collected data was identified as a barrier. Moves to make data findable, accessible, interoperable, and reusable (FAIR) have been promoted as a means to encourage greater accessibility to data in a systematic way [38]. The FAIR principles focus on data characteristics and should be interpreted alongside the collective benefit, authority to control, responsibility, and ethics (CARE) principles established by the Global Indigenous Data Alliance (GIDA) which a people and purpose orientated [39].

### Limitations

The papers included in this study were limited to those indexed on major databases. Some literature on this topic may have been excluded if it was not identified during the grey literature and hand searching phases.

### Implications

Results of this systematic literature review indicate that while there is broad agreement for the principles of data sharing in medical research, there remain disagreements about the infrastructure and procedures associated with the data sharing process. Additional work is therefore required on areas such as acknowledgement, curation, and data ownership.

## Conclusion

While the literature confirms that there is overall support for data sharing in medical and scientific research, there remain significant barriers to its uptake. These include concerns about privacy, consent, information security, and data ownership.

## Data Availability

All data generated or analysed during this study are included in this published article.

## Abbreviations

BRIF: Bioresource Research Impact Factor/Framework
CARE: Collective benefit, authority to control, responsibility, and ethics
CoBRA: Citation of BioResources in journal Articles
FAIR: Findable, accessible, interoperable, and reusable
GIDA: Global Indigenous Data Alliance
HIV/AIDS: Human immunodeficiency virus/acquired immunodeficiency
ICMJE: International Council of Medical Journal Editors
MS: Multiple sclerosis
SEER: Surveillance, Epidemiology, and End Results
TB: Tuberculosis
TCGA: The Cancer Genome Atlas
